# Towards smart self-clearing glaucoma drainage device

**DOI:** 10.1038/s41378-018-0032-3

**Published:** 2018-11-05

**Authors:** Hyunsu Park, Amir Hossein Raffiee, Simon W. M. John, Arezoo M. Ardekani, Hyowon Lee

**Affiliations:** 1 Weldon School of Biomedical Engineering, Birck Nanotechnology Center, Center for Implantable Devices, Purdue University, West Lafayette, IN 47907 USA; 2 School of Mechanical Engineering, Purdue University, West Lafayette, IN 47907 USA; 3 Howard Hughes Medical Institute, The Jackson Laboratory, Bar Harbor, ME 04609 USA

## Abstract

For patients who are unresponsive to pharmacological treatments of glaucoma, an implantable glaucoma drainage devices (GDD) are often used to manage the intraocular pressure. However, the microscale channel that removes excess aqueous humor from the anterior chamber often gets obstructed due to biofouling, which necessitates additional surgical intervention. Here we demonstrate the proof-of-concept for smart self-clearing GDD by integrating magnetic microactuators inside the drainage tube of GDD. The magnetic microactuators can be controlled using externally applied magnetic fields to mechanically clear biofouling-based obstruction, thereby eliminating the need for surgical intervention. In this work, our prototype magnetic microactuators were fabricated using low-cost maskless photolithography to expedite design iteration. The fabricated devices were evaluated for their static and dynamic mechanical responses. Using transient numerical analysis, the fluid–structure interaction of our microactuator inside a microtube was characterized to better understand the amount of shear force generated by the device motion. Finally, the anti-biofouling performance of our device was evaluated using fluorescein isothiocyanate labeled bovine serum albumin. The microactuators were effective in removing proteinaceous film deposited on device surface as well as on the inner surface of the microchannel, which supports our hypothesis that a smart self-clearing GDD may be possible by integrating microfabricated magnetic actuators in chronically implanted microtubes.

## Actuators: Glaucoma drainage devices

The addition of a magnetically powered microdevices inside the tube of a glaucoma drainage device enables the removal of debris caused by biofouling without surgery. Patients suffering from glaucoma often require the insertion of a drainage device to remove excess aqueous humor from the eye, so that the intraocular pressure does not reach a level that could damage the optic nerve. However, the microtubes that these devices contain often become blocked as a result of biofouling, requiring the patient to undergo an operation. Now, a team led by Hyowon Lee from Purdue University, USA, integrate microactuators that can be activated using an externally applied magnetic field, removing the build-up of debris without invasive surgery.

## Introduction

Glaucoma is a group of eye diseases that causes progressive damage to optic nerve. It is commonly known as “the silent thief of sight” due to the lack of symptoms during the early stages^1^. Because of this difficulty in early diagnosis, glaucoma remains as one of the leading causes of blindness and visual impairments in the world^2^. It currently affects around 64.3 million people in the world and this number is expected to double by 2040^3,4^. In the United States, there are more than 3 million patients with glaucoma and it disproportionally affects African Americans and Hispanics^5–9^. Glaucoma is a major healthcare issue with the annual cost for treatment in the US that exceeds $2.9 billion^10^.

Typically, glaucoma patients experience poor drainage of aqueous humor (AH) through the natural outflow pathways (i.e., trabecular meshwork and Schlemm’s canal)^11^. The imbalance between the rate of production and the outflow of AH from the eye causes an increased intraocular pressure (IOP), which is a major risk factor that leads to subsequent damage to optic nerve and the loss of eyesight^12,13^. Unfortunately, there is no cure for glaucoma. However, the progression of disease can significantly be delayed using pharmaceutical and surgical interventions that maintain the IOP in a safe range to minimize optic nerve damage^14^. Glaucoma drugs are typically designed to decrease the production of AH or to increasing its outflow through trabecular meshwork or uveoscleral pathway^15–18^. As with most pharmaceutical interventions, however, these drugs have several undesirable side effects including bitter taste, headache, conjunctivitis, visual blurring, eyelid inflammation, and eye pain^19–21^. Surgical treatments such as trabeculectomy and laser trabeculoplasty can also be used to increase AH outflow but these invasive procedures often lead to serious post-operative complications such as hypotony, cataract, and bleb-related infections^22–25^. Moreover, the surgical ablation of trabecular meshwork often results in coagulative necrotic tissue, which can cause difficulty in chronic management of optimal AH outflow^26^.

For patients with refractory or inflammatory glaucoma who are unresponsive to conventional pharmacological or surgical procedures, glaucoma drainage device (GDD) are often implanted. These devices offer several advantages over conventional trabeculectomy including better IOP control, ease of surgery, and minimum post-surgical complications^27–29^. Traditional GDDs consists of a short polymeric microscale tube that connects the anterior chamber to a thin silicone plate for drainage of excess AH^30–32^. While GDDs have been used to manage IOP for glaucoma patients for the past 40 years, 15.1% of implanted devices fail within 3 years and more than 29.8% fail within 5 years post-implantation^33,34^. Clinical studies have shown that up to 10% of glaucoma patients require additional medications and surgical intervention because of the tube blockage^35^. The hydrophobic polymer materials from which GDDs are constructed (e.g., polypropylene, polymethylmethacrylate, and polydimethylsiloxane) typically have high affinity for interstitial proteins such as fibrinogen, immunoglobulin, and albumin that adsorbs onto the device surface within minutes after the implantation^36–38^. Once it forms, the proteinaceous layer triggers the inflammatory response that can lead to premature implantable device failure^39^. Since GDDs generally have a drainage tube with an inner diameter that ranges from 50 to 600 μm, the microscale channel can easily be occluded by various biofouling materials including vitreous, fibrin, or blood clot^40–43^.

One promising approach that can remove adsorbed biofouling material is to ablate occlusion using neodymium-doped yttrium aluminum garnet (Nd:YAG) laser. However, there are several potential risks associated with laser treatments including focal cataracts, prolonged elevation of the intraocular pressure, posterior capsule rupture, retinal injury, and laser injury^44–50^. Tissue plasminogen activator (tPA), which is a serine protease involved in the breakdown of fibrin or blood clots, has also been used to clear occluded glaucoma shunts^51,52^. However, tPA may cause additional undesirable complications such as hyphema, active bleeding, and vitreous hemorrhage^53–55^.

**Fig. 1:**
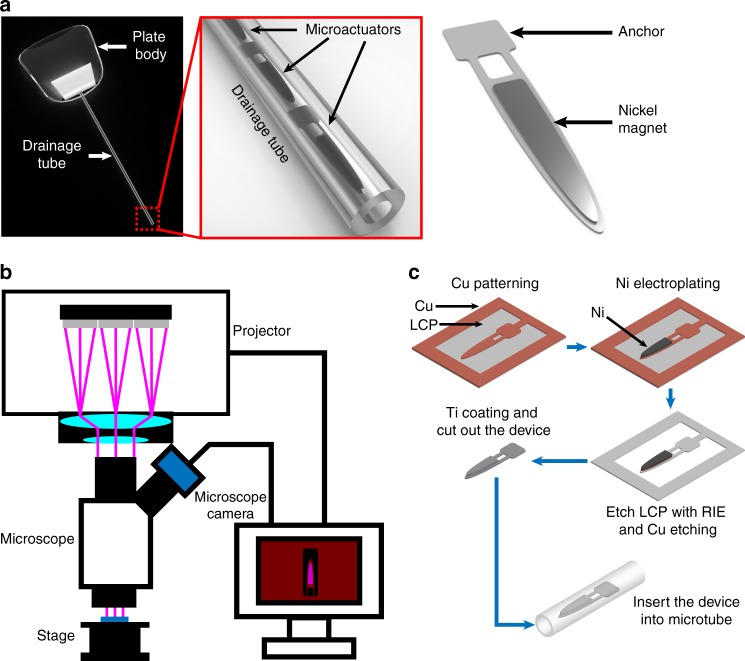
Microactuator based self-cleaning GDD. **a** 3D schematics of self-clearing GDD. **b** A custom maskless photolithography setup. **c** Fabrication procedure for the magnetic microactuator

Establishing a method to non-invasively remove biofouling without causing side effects can significantly improve the reliability and functionality of many chronically implanted devices. Here we report on the design, fabrication, and testing of anti-biofouling microtube integrated with an array of magnetic microactuators as a part of a self-clearing GDD that can actively combat against proteinaceous biofouling in situ without the need for additional surgical or pharmaceutical interventions. We believe our strategy to remove bioaccumulation on-demand using externally applied magnetic field is a way to significantly improve the functional lifetime of implantable devices that suffer from biofouling-related performance degradation. By integrating thin-film magnetic microactuators fabricated out of liquid crystal polymer (LCP) using maskless lithography, here we demonstrate a low-cost prototype of self-clearing GDD drainage tube (Fig. 1). Using fluorescent-tagged bovine serum albumin, we show the protein-clearing capabilities of these prototype GDD microtubes using time-varying magnetic fields, which may eliminate the need for additional surgical or pharmaceutical interventions for glaucoma patients.

## Results

### Device fabrication and mechanical characterization

**Fig. 2:**
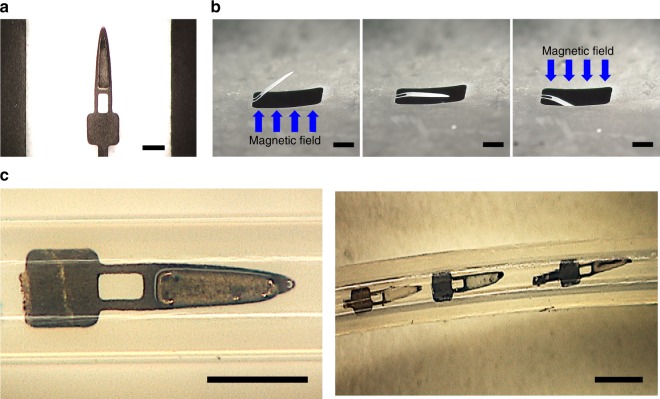
Images of fabricated microactuators and self-clearing GDD. **a** Digital photograph of the fabricated magnetic microactuators. Scale bar = 200 μm **b** The deflected microactuator with different directional magnetic flux density with 13.7 mT. Scale bar = 200 μm. **c** Digital photographs of the integrated microactuators in the lumen of a prototype GDD microtube. Scale bar = 500 μm

Figure 2a shows our microfabricated LCP-based device. The needle-shape was chosen to accommodate the relatively small tube diameter. We can control the deflection direction and amplitude of the microactuator by adjusting the strength and the direction of the externally applied magnetic field (Fig. 2b). We assembled the microdevices into a prototype GDD drainage tube using an anchor to demonstrate protein-removal performance inside the tube (Fig. 2c). Once manually placed into the microtube, we heated the tube and applied tensile stress at both ends to decrease the diameter of the microtube and fix the microactuators in position, which prevented any shifting of devices during actuation in a continuous fluid flow.

To characterize the actuation capabilities of our magnetic microactuators, we evaluated the static and dynamic mechanical responses. A magnetic moment of the soft ferromagnetic element is generated when the magnetic microactuator is placed in a static magnetic field. The microactuators can deflect out of plane when the direction of the applied magnetic field is normal to the magnetization direction of the ferromagnetic element (Fig. 2b). The deflection angle of magnetic microactuator can be described in the ref. ^56^

1 $$\phi = \frac{{V_{\mathrm{m}}\left( {\vec M \times \vec H} \right)}}{{k_{{\mathrm{beam}}}}}$$

with the angular deflection *ϕ*, magnet volume *V*_m_, magnetization $\vec M$, applied magnetic field $\vec H$, and the flexure stiffness *k*_beam_. The beam geometry and the material property affect the mechanical stiffness of the flexure with following

2 $$k_{{\mathrm{beam}}} = \frac{{E_{\mathrm{c}}wt^3}}{{12l}}$$

with the elastic modulus *E*_c_, beam width *w*, beam thickness *t*, and beam length *l*^57,58^.

**Fig. 3:**
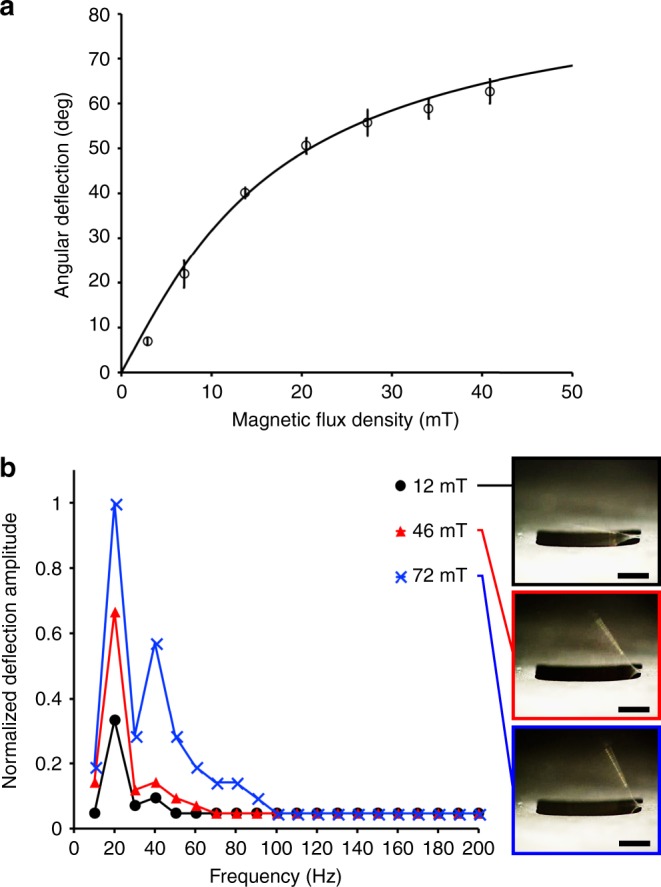
Static and dynamic characterization. **a** Theoretical and measured angular deflections (*n* = 3). **b** Frequency response of magnetic microactuator in the water. Note the captured images of microactuators in resonance at various magnetic flux density levels (inset). Scale bar = 500 μm

As shown in Fig. 3a, the measured deflection angle corresponded closely with the theoretical values. We varied the frequency and amplitude of the externally-applied, time-varying magnetic fields using a custom electromagnet to obtain the frequency response (10–200 Hz) of our microactuators (Fig. 3b). As expected, the amplitude of deflection increased as a function of applied magnetic field strength. Furthermore, we determined that the actuation frequency of 20 Hz to be the primary resonance, which can be used to generate the highest dynamic deflection amplitude. The increase in dynamic deflection amplitude may be attributable to the increase in mean fluid velocity around the microactuator, which leads to an increase in the wall shear stress on the microactuator and the tube^59,60^. Therefore, we used a fixed actuation frequency (20 Hz) with the highest actuation amplitude (64°) for all experiments and simulation.

To verify these Ti-coated LCP based microactuator is robust enough to withstand a large number of actuation cycles in physiological condition, we examined the changes in the dynamic responses of these microdevices in 37 °C phosphate buffered saline (PBS, ThermoFisher Scientific, Waltham, MA, USA). After 10.9 million actuation cycles, we saw no visible damage to the LCP-based microactuators and no change in the resonant frequency of tested devices (*n* = 4, Supplementary Fig. 1). If we assume a 5-min weekly actuation, this equates to up to 35 years of lifetime, which suggests adequate robustness for our LCP-based microactuators against fatigue related failure.

### Fluid–structure interaction

**Fig. 4:**
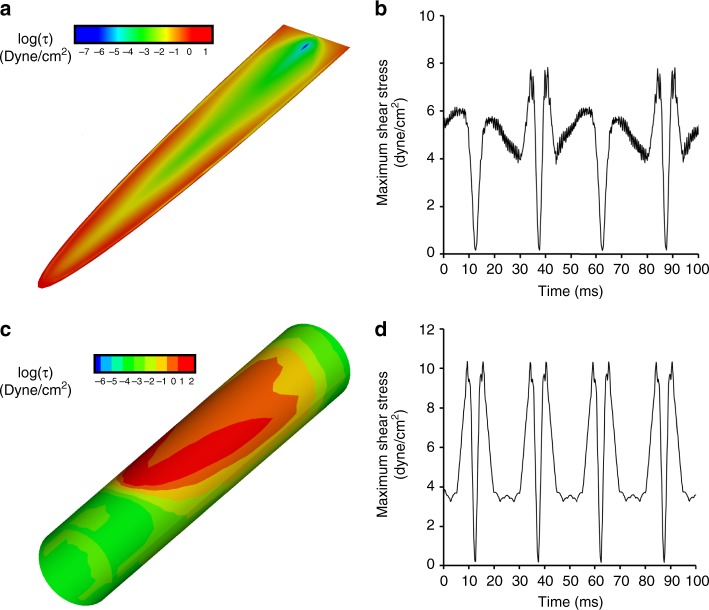
Numerical analysis of shear stress distribution. **a** Shear stress distribution generated on the surface of the actuators. **b** Maximum shear stress on the actuator surface as a function of time. **c** Shear stress distribution generated on the tube. **d** Maximum shear stress from the internal surface of the microtube as a function of time

We evaluated the shear stress distribution generated by the microactuation motion using finite element modeling. The simulation results showed that the maximum shear stress is generated near the perimeter of the actuator (Fig. 4a). When the device is integrated into the microtube, the actuation leads to a larger shear stress as the microactuator approaches the tube wall (Fig. 4c). During the actuation, the maximum shear stress of ~8 and ~10 dyn/cm^2^ are generated periodically on the surface of the actuator and the tube, respectively (Fig. 4b, d).

### BSA-FITC adsorption and desorption

**Fig. 5:**
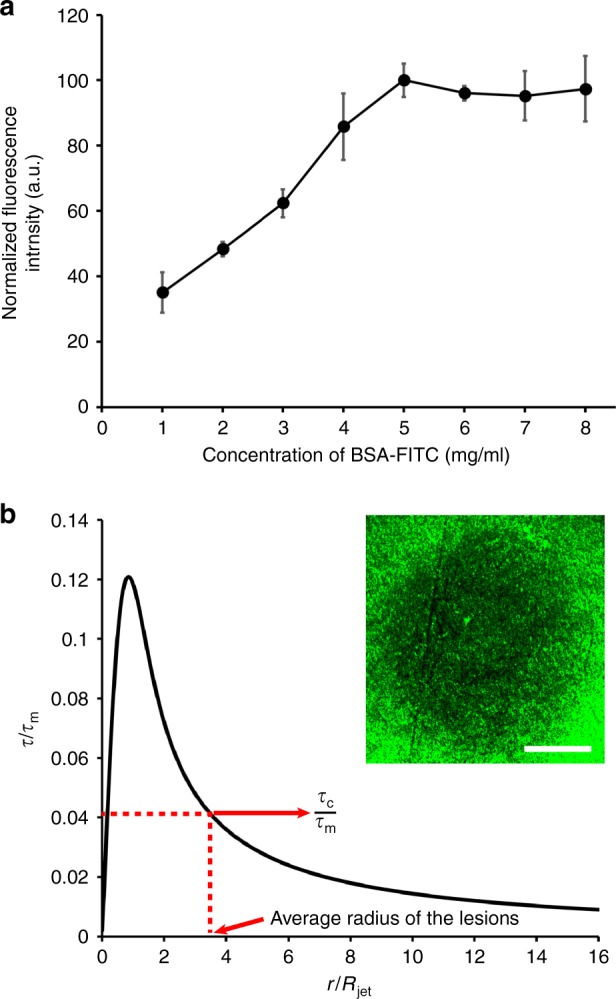
Optimization of BSA-FITC coating and jet impingement test. **a** The relationship between adsorbed BSA-FITC and the concentration of the protein solution with 2 h incubation (n = 5). **b** Plot of non-dimensional shear stress versus lesion size. Note the image of lesion created using jet impingement (inset). Scale bar = 200 μm. The dotted line and arrows point to the average lesion size and the corresponding non-dimensional shear stress

To maximize the fluorescence intensity, we incubated Ti-coated LCP samples in various concentrations of BSA-FITC (1–8 mg/ml) for 2 h. The fluorescence intensity of absorbed BSA-FITC plateaued around 5 mg/ml (Fig. 5a), therefore, all subsequent BSA-FITC evaluations used this concentration. The jet impingement technique is widely used to analyze the shear stress required to remove cells by corresponding the size of a lesion created by a perpendicular jet of fluid to a well-characterized shear stress profile^61,62^. To quantify the adhesion strength of BSA-FITC on Ti-coated LCP surface, we used the theoretical description of the wall shear stress under the impinging jet proposed by Phares et al.^63^. For this analysis, we assumed that the AH is incompressible Newtonian fluid in a steady and laminar flow. In the theoretical description of the wall shear stress in normally impinging jet with jet height *H*, the wall shear stress *τ* at a radial distance *r* can be described by

3 $$\frac{\tau }{{\tau _{\mathrm{m}}}} = 0.18\left( {\frac{{1 - e^{ - 114\lambda ^2}}}{\lambda }} \right) - 0.943\lambda e^{ - 114\lambda ^2}$$

with the maximum shear stress *τ*_m_ and non-dimensionalized jet height (*λ* = *r*/*H*). The maximum shear stress *τ*_m_ is given by

4 $$\tau _{\mathrm{m}} = 0.16\frac{{\rho u_{\mathrm{o}}^2}}{{(H{\mathrm{/}}D)^2}}$$

with the fluid density *ρ*, the average flow velocity at the nozzle exit *u*_o_, and diameter for the nozzle *D*. The critical shear stress (*τ*_c_) required to remove the adsorbed BSA-FITC can then be calculated by measuring the radius of lesion (Fig. 5b).

In the jet impingement test, the fluid jet was delivered at a flow rate of 1.18 ml/min for 5 s, which corresponds to Reynolds number of 100 in laminar flow range to be used for Eqs. (3) and (4). The fluid jet created *τ*_m_ ~ 30 dyn/cm^2^ which is in line with published shear stress value required to rupture protein-ligand interaction^64^. Figure 5b shows an image of BSA-FITC lesion created by jet impingement and a plot of non-dimensional wall shear stress as a function of non-dimensional lesion size for jet radius *R*_jet_ = 125 μm. With an average lesion radius of 284 μm (n = 4), the estimated shear stress required to remove BSA-FITC (*τ*_c_) was 10.2 dyn/cm^2^. The numerical analysis results (Fig. 4) showed that our magnetic microactuators can generate up to 10 dyn/cm^2^. Taken together with the results from our jet impingement study, we expected to show a robust protein removal using our prototype GDD drainage tube.

### Protein biofouling removal in GDD

The main function of our magnetic microactuators is to remove the protein adsorbed on the device surface and the inner wall of GDD microtube to prevent the initiation of inflammatory cascade. As such, we quantified the decrease in fluorescence intensity due to device actuation on device surface and the inner wall of the microtube as simulated in Fig. 4. To study anti-biofouling capability of the actuator itself, we actuated BSA-FITC coated devices with different actuation durations at 20 Hz. The maximum actuation duration was set to 5 min based on prior literature^65,66^ and for practical consideration assuming that a shorter actuation protocol would be less burdensome on clinicians and patients. The minimum actuation duration was set to be 30 s, which is 10% of the maximum actuation duration.

**Fig. 6:**
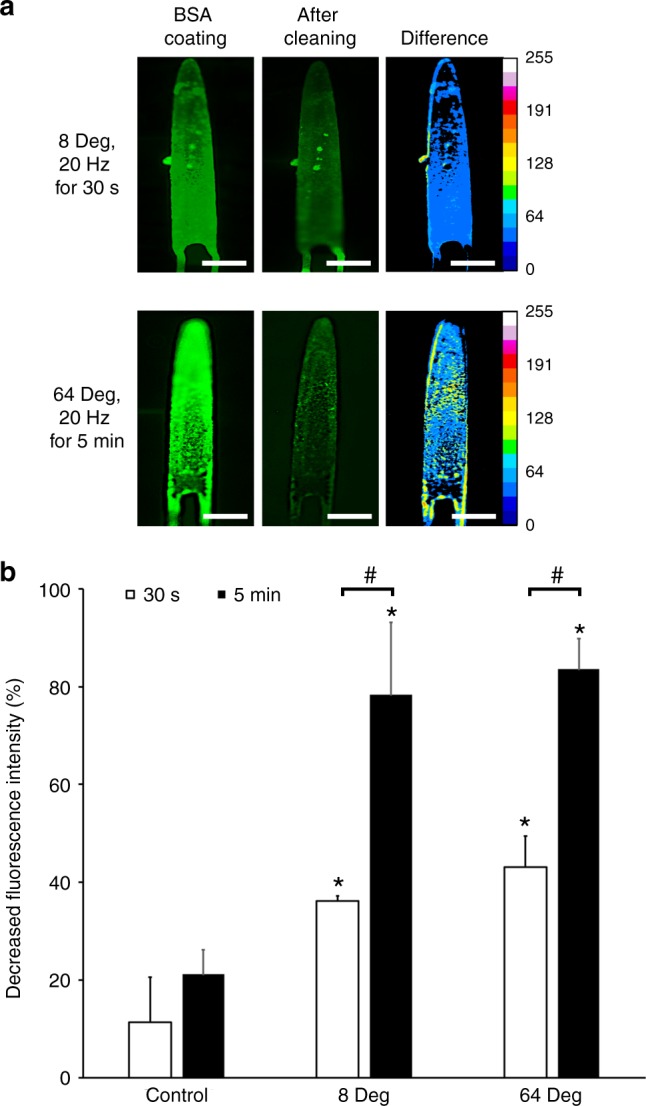
Impact of actuation amplitude and duration. **a** Fluorescence intensities (a. u.) of BSA-FITC coated microactuators before and after actuation using different deflection amplitudes and actuation duration at 20 Hz. Significant protein removal can be seen for both small and large amplitude actuation. The difference map shows a more significant protein removal with longer actuation duration. Scale bar = 200 μm. **b** Comparison of decreased fluorescence intensity (*n* = 3 for each actuation condition). * and # indicates statistical significance against corresponding control (*p* < 0. 01)

Figure 6 demonstrates BSA-FITC removal due to actuation of magnetic microdevices. We compared the decreased fluorescence intensity values using one-way analysis of variance (ANOVA) with Tukey’s HSD post-hoc pairwise analysis. The results showed that BSA-FITC coated on microactuators (*n* = 3) was significantly reduced compared to non-actuated control regardless of deflection amplitude or actuation duration (p < 0.01). Without actuation, the fluorescence intensity decreased by approximately 10–20% depending on treatment duration. However, the difference in fluorescent intensity between the small (8°) and large (64°) deflection magnitudes was not statistically significant. The impact of actuation duration was also statistically significant. When actuated for 30 s, BSA-FITC amount reduced by 42%. whereas up to 85% protein clearance can be seen on device when actuated for 5 min. Thus, in subsequent evaluations to determine the impact of actuation on removing protein adsorbed on the microtube inner wall, we actuated all samples for 5 min to maximize protein clearance.

**Fig. 7:**
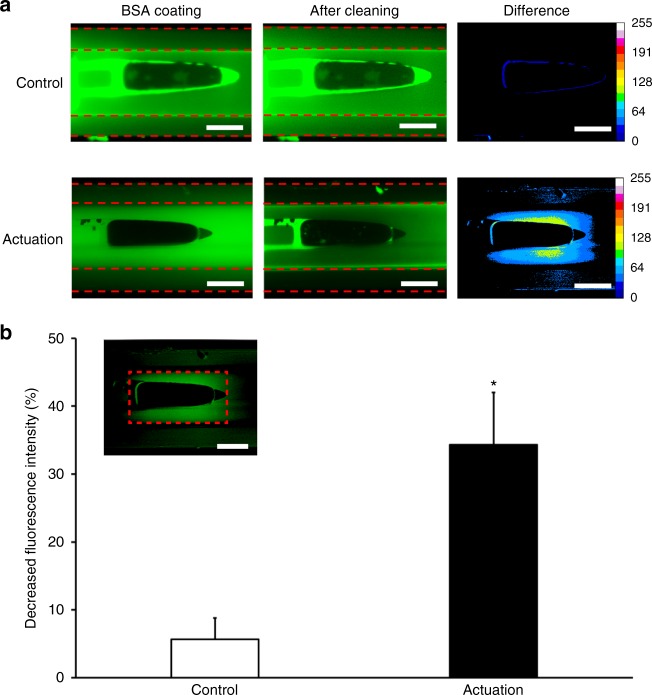
Protein cleaning in the microtube. **a** Fluorescence images of BSA-FITC removal using the microactuator located within the lumen of microtube with an actuation for 5 min at 20 Hz (a. u., scale bar = 200 μm). **b** The area used for measuring fluorescence intensity (red dotted box (scale bar = 200 μm) and decreased fluorescence intensity near the actuator

To demonstrate the in situ anti-biofouling performance of our smart GDD, we coated the inner lumen of 300-μm-diameter microtube integrated with our magnetic microactuator using BSA-FITC (Fig. 2c). Figure 7a highlights the difference in fluorescence intensity between actuated versus non-actuated GDD prototype. Without actuation, we saw virtually no difference in fluorescence intensity. Following actuation, however, we saw a significant decrease in fluorescence intensity in areas surrounding the microdevice. The pattern of cleared area closely resembles the shear stress distribution predicted by our numerical analysis (Fig. 4). We then quantified the amount of fluorescence intensity decrease from the end of the beams to the actuator tip and compared the results using a two-sample *t*-test. The results show that the microactuation can remove significant amount of adsorbed BSA from the tube wall compared to the non-actuated control (*p* < 0.01, Fig. 7b). However, the decrease in fluorescence intensity (<40%) in microtube was much smaller than the 85% decrease we saw from the device surface following a 5 min actuation (Fig. 6b).

## Discussion

Here we demonstrated that the LCP-based microactuators can easily be fabricated at low-cost using our maskless photolithography. LCP is widely used polymer in biomedical applications due to their near hermetic properties, biocompatibility and superior chemical resistance^67–70^. By using commercially available low-cost LCP sheets as the substrate in combination of highly scalable microfabrication processes such as maskless photolithography, oxygen etching, electroplating, and polymer coating, it is possible to manufacture these LCP-based devices at extremely low costs. Moreover, the simple integration process that we employed to immobilize microdevices inside a small drainage tube may be used to create other smart MEMS-enabled catheter-based devices.

Both the static and the dynamic responses of these microfabricated LCP-based actuators corresponded well with the theoretical values, which suggests a good control of our fabrication process. The in vitro evaluation using BSA-FITC showed that, as expected, the actuation from our device can effectively reduce proteinaceous biofouling on the actuator surface and the inner wall of the microtube. The results from the in vitro experiments demonstrated a good agreement with our results from the numerical analysis that predicted the pattern of biofilm clearance by quantifying the shear stress distribution and the jet impingement study that quantified the adhesion strength of the BSA-FITC. This approach to quantify the adhesion properties of specific biofilm and to model the shear stress profile of a device actuation may be used in future iterations to design novel microactuator arrays that are tailored for bespoke implantable application against specific biofouling materials.

To confirm that the biofouling removal process does occur via mechanical shear generated by the microactuation and not by the heat generated from the microactuation, we also measured the amount of heat generated during actuation (Supplementary Fig. 2). When we actuated our microdevices (*n* = 4) for 5 min at 20 Hz using 40 mT in room temperature PBS, no temperature increase was seen in thermal camera images (FLIR A325sc, FLIR, Wilsonville, OR, USA). It is important to ensure that no thermal effect occurs due to microactuation since excessive heat may lead to unintended damage to the surrounding tissue.

The potential implication of utilizing active mechanism for combating biofouling is enormous since many chronically implantable devices including biosensors, neural interface electrodes, and drug delivery and drainage devices suffer from significant performance degradation due to biofouling^39,71^. Although there is a number of proposed mechanisms for actively addressing biofouling using electrical and mechanical transducers^65^, the magnetically-powered actuators have several key advantages. First, the magnetic device can be activated in situ wirelessly via externally applied magnetic field with low power requirements (Supplementary Fig. 3) without the need for any invasive procedure. Second, the magnetic microactuators can be tailored to deliver large disruptive forces to remove multi-scale biofouling materials including protein, bacteria, and cells. Third, the lack of integrated circuit and internal power source can facilitate the integration and packaging of these type of devices into existing medical devices, which can accelerate clinical adoption. Finally, as mentioned, the simple design is compatible with many scalable microfabrication technologies that can significantly reduce the cost of manufacturing.

Despite these key benefits, there are several remaining questions to be answered. First, the amount of protein removed from the microtube wall was much lower than that from the actuator surface despite our numerical analysis demonstrating a higher maximum shear stress on the wall. This may be due to the fact that each microscope image was focused on microactuator surface, which is located at the center of the microtube. As can be seen from Fig. 4 and the Supplementary Video, the magnitude of shear stress distribution around the mid-plane of microtube is much lower than the top and bottom of the microtube. It may be interesting to characterize protein distribution using a confocal microscope in the future to verify this hypothesis. If not, it is possible to leverage our predictive modeling to redesign microactuators that can provide a greater average shear stress to ensure a more efficient protein-removal. Secondly, additional experiments are needed to determine optimum actuation duty cycle that will ensure a protein-free GDD microtube. Although the microactuators were able to demonstrate good protein reduction in just 5 min, it may be possible to reduce this actuation duration further by performing a systematic evaluation. Thirdly, although it is possible to integrate many microactuators into a long microtube (Supplementary Fig. 4), the manual assembly process can be further streamlined if 1D arrays of microactuators are fabricated to better control the device spacing. Finally, additional in vitro and in vivo work is necessary to ascertain whether periodically removing biofilm using our self-clearing implants will actually prolong the device lifetime and improve patient outcome. A critical question to address is to determine what happens with the displaced biofouling material. A detailed histopathological evaluations using animal models must be performed to ensure that the displaced biomaterial will not cause undesirable downstream effects.

## Materials and methods

### Device fabrication

We fabricated the microactuators from copper-(Cu)-cladded LCP using a custom maskless photolithography setup previously described^72^. We used a computer connected to a conventional home theater projector with a digital micromirror device (HD142X, Optoma, Fremont, CA, USA) to project and expose a desired pattern^72–75^. The projector was vertically fixed on a stereo-microscope (SM-4B, Amscope, Irvine, CA, USA) using a custom machined bracket. To improve the resolution and reduce the size of the image, we optimized the alignment between the lens of the microscope, the sample stage, and the projector. We used Microsoft PowerPoint to design and project various mask patterns. We adjusted the exposure intensity by modifying pattern color in the software. Figure 1c shows the overall process flow for the device fabrication.

The commercially available LCP sheet (Ultralam 3850, Rogers corporation, Chandler, AZ, USA) has a thickness of 25 μm. To improve compliance of the cantilevers, we reduced the LCP thickness to 8 μm using a reactive ion etcher (RIE, PlasmaPro80, Oxford Instruments plc, Abingdon, Oxfordshire, United Kingdom) after removing Cu from one side using a wet Cu etchant (CE-100, Transene, Danvers, MA, USA). We then mounted the 8-μm-thick single clad LCP sheet onto a carrier wafer using a positive photoresist (PR) (AZ9260, Microchem, Westborough, MA, USA) with the Cu on top. We spin coated AZ9260 onto the Cu layer and exposed the cantilevers designs using our custom maskless photolithography setup. After etching the Cu layer using a wet Cu etchant (CE-100, Transene, Danvers, MA, USA), we removed PR using acetone. Next, we defined the Ni magnet electroplating mold on spin-coated AZ9260 using the same maskless photolithography procedure. We electroplated Ni to achieve a final thickness of 20 μm. After removing the PR, we etched the cantilever pattern on bare LCP layer using an RIE and removed the remaining Cu layer using a chemical etchant (BTP, Transene, Danvers, MA, USA). Finally, we coated the device with 100 nm thick titanium (Ti) using a sputterer (Magnetron sputtering systems, PVD Products, Inc., Wilmington, MA, USA) on both sides to improve biocompatibility.

### Mechanical characterization

We measured the magnitudes of angular deflections for a range of applied magnetic flux density up to 40.9 mT. We used a bespoke iron-core electromagnet to generate the magnetic field. We quantified the strength of magnetic field using a commercial gaussmeter (Model 8010, Pacific Scientific OECO, Milwaukie, OR, USA). We then placed the device on top of the electromagnet and applied the magnetic field of varying amplitude and frequency. We imaged the deflected actuators using a digital microscope KH8700, Hirox, Hackensack, NJ, USA) and calculated the deflection angles from the images using imageJ software (version 1.50i). To better characterize the motion of the devices in liquid, we also characterized the dynamic responses of the magnetic microactuators in deionized water using a custom laser deflecting setup. Using a mirror, we placed a laser beam onto the metallic surface of the device, which then reflected the laser beam onto a position sensitive diode (PSD) sensor. We recorded the two-dimensional position data from the PSD using a custom data acquisition system (LabView 2014, Austin, TX, USA).

### Fatigue evaluation

As a baseline, we photographed and measured the dynamic responses of each test sample (*n* = 4). We immobilized each microactuator on glass slide using polyimide tape for the fatigue evaluation. We then placed the glass slide fixture in a beaker filled with PBS at 37 °C. We actuated the device for 6 days to achieve 10.9 million cycles at 12 mT and 20 Hz sinusoidal signal. Following the continuous actuation, the microactuators were removed from the beaker, photographed, and analyzed for post-actuation dynamic response.

### Fluid–structure interaction

To determine the shear stress generated by the actuation, we used a finite volume method to simulate shear stress on the surface of microactuator and the lumen of the microtube by numerically solving Navier–Stokes equations. We discretized the computational domain using a uniform, staggered, cartesian grid. We used Euler explicit method for time discretization and spatial derivatives in convective and computed diffusive terms using the quadratic upstream interpolation for convective kinematics and central difference schemes, respectively^76^. Furthermore, we coupled the pressure and velocity using a projection method^77^. We implemented a distributed Lagrange multiplier method to simulate the motion of microactuator in a viscous fluid, which allowed us to accurately capture the hydrodynamic interaction between the microactuator and a surrounding fluid and evaluate the shear stress acting on the surface^78,79^.

### Protein-based biofouling adhesion

We tested the anti-biofouling performance of our magnetic microactuators using fluorescent-tagged bovine serum proteins (BSA-FITC, ThermoFisher Scientific, Waltham, MA, USA), which is readily coated onto the implant surface via non-specific binding and subsequently initiates the inflammatory response in vivo^80^. We incubated the devices and samples for jet impingement test in the BSA-FITC solution of various concentrations (1–8 mg/ml) in PBS (*n* = 5, each) for 2 h and rinsed with deionized water. We captured the images of protein coated samples using a fluorescence microscope (Axio Observer Z1, Carl Zeiss Microscopy, LLC) and a filter set 17 (excitation, BP 485/20, and emission BP 515-565, Carl Zeiss Microscopy, LLC), and quantified the fluorescence intensity using imageJ. We normalized each image using the imageJ against bare non-coated sample.

We quantified the magnitude of shear stress required to remove the absorbed protein on Ti-coated surface using a jet impingement experiment. We vertically placed the tip of a 15-ml syringe with the needle having an inner diameter of 250 μm (7018333, Nordson EFD, East Providence, RI, USA) 1 mm over BSA-FITC coated substrate and delivered the jet flow using a syringe pump (NE-300, New Era Pump Systems, Inc., Farmingdale, NY, USA). We created a total of four lesions. Using the same fluorescence microscope describe above, we imaged of each lesion and measured the diameter of each using imageJ. We subtracted the background fluorescence from bare substrate to normalize fluorescence intensity,

### Protein biofouling removal in GDD

We investigated the impact of deflection amplitude (8° vs. 64°) and actuation duration (30 s vs. 5 min) of our magnetic microactuators by quantifying the amount of BSA-FITC (*n* = 3, each). We placed each sample in deionized water in a custom testing chamber to block the ambient light during actuation. We captured the fluorescence images of protein-coated devices before and after the actuation and quantified the difference in fluorescence intensity using imageJ. We normalized the background fluorescence by subtracting fluorescencefrom a bare actuator surface. To demonstrate the anti-biofouling capability of magnetic microactuators inside a polytetrafluoroethylene microtube, we coated the lumen of assembled GDD drainage tube by flowing BSA-FITC at 2.7 μl/min, which is the average flow rate of AH in human eyes. We then actuated the microdevices at 20 Hz for 5 min to remove the adsorbed protein layer (*n* = 3). Finally, we quantified the decrease in fluorescence level due to actuation and compared with the non-actuated controls.

## Electronic supplementary material

Supplementary Figures

Video of self-clearing GDD microtube

Shear stress distribution on device surface during actuation

Shear stress distribution on inner surface of microtube during actuation
