# Supplementary material for: Towards smart self-clearing glaucoma drainage device
Source: Microsyst Nanoeng. 2018 Nov 5;4:35. doi: 10.1038/s41378-018-0032-3 (PMC6220179; doi:10.1038/s41378-018-0032-3)
Supplement: Supplementary file 1 — Supplementary Figures [file 41378_2018_32_MOESM1_ESM.pdf]

Towards Smart Self-Clearing Glaucoma Drainage Device

Hyunsu Park[1], Amir Hossein Raffiee[2], Simon W. M. John[3], Arezoo M. Ardekani[2], and Hyowon Lee[1,*]

[1]Weldon School of Biomedical Engineering, Birck Nanotechnology Center, Center for Implantable Devices, Purdue University, West Lafayette, IN, USA
[2]School of Mechanical Engineering, Purdue University, West Lafayette, IN 47907, USA
[3]Howard Hughes Medical Institute, The Jackson Laboratory, Bar Harbor, ME 04609, USA
[*]Correspondence to hwlee@purdue.edu

ABSTRACT

Supplementary Information

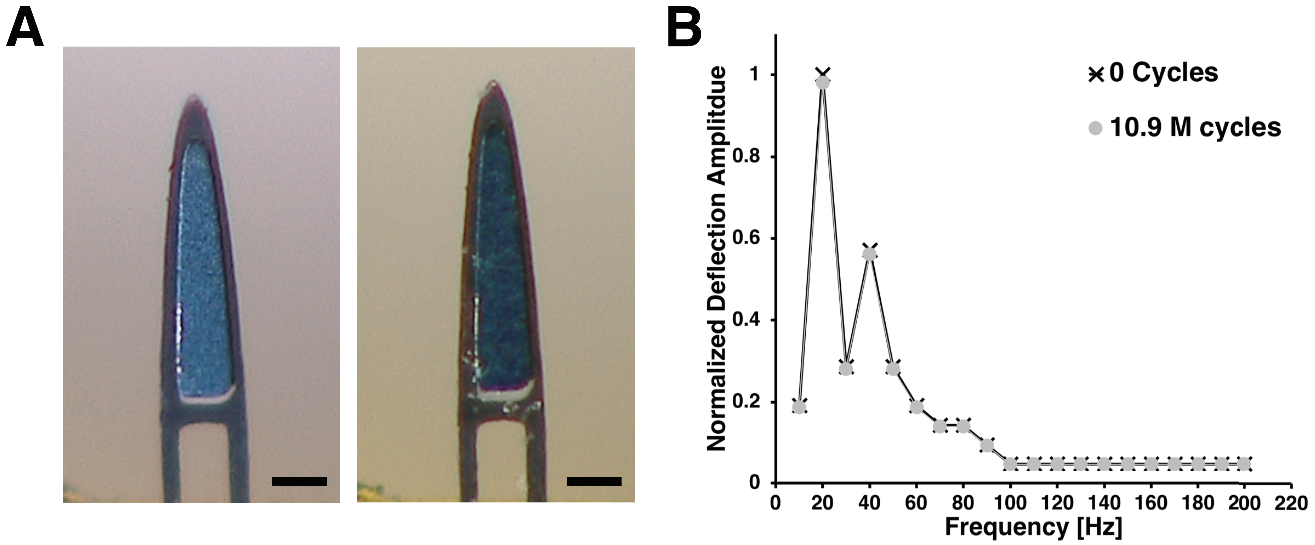

Figure 1. Fatigue evaluations. (A) Photographs of a microactuator before actuation (left) and after 10.9 M actuation cycles (right). Scale bar = 100 μm. (B) Frequency responses of a representative device before and after long-term actuation in PBS.

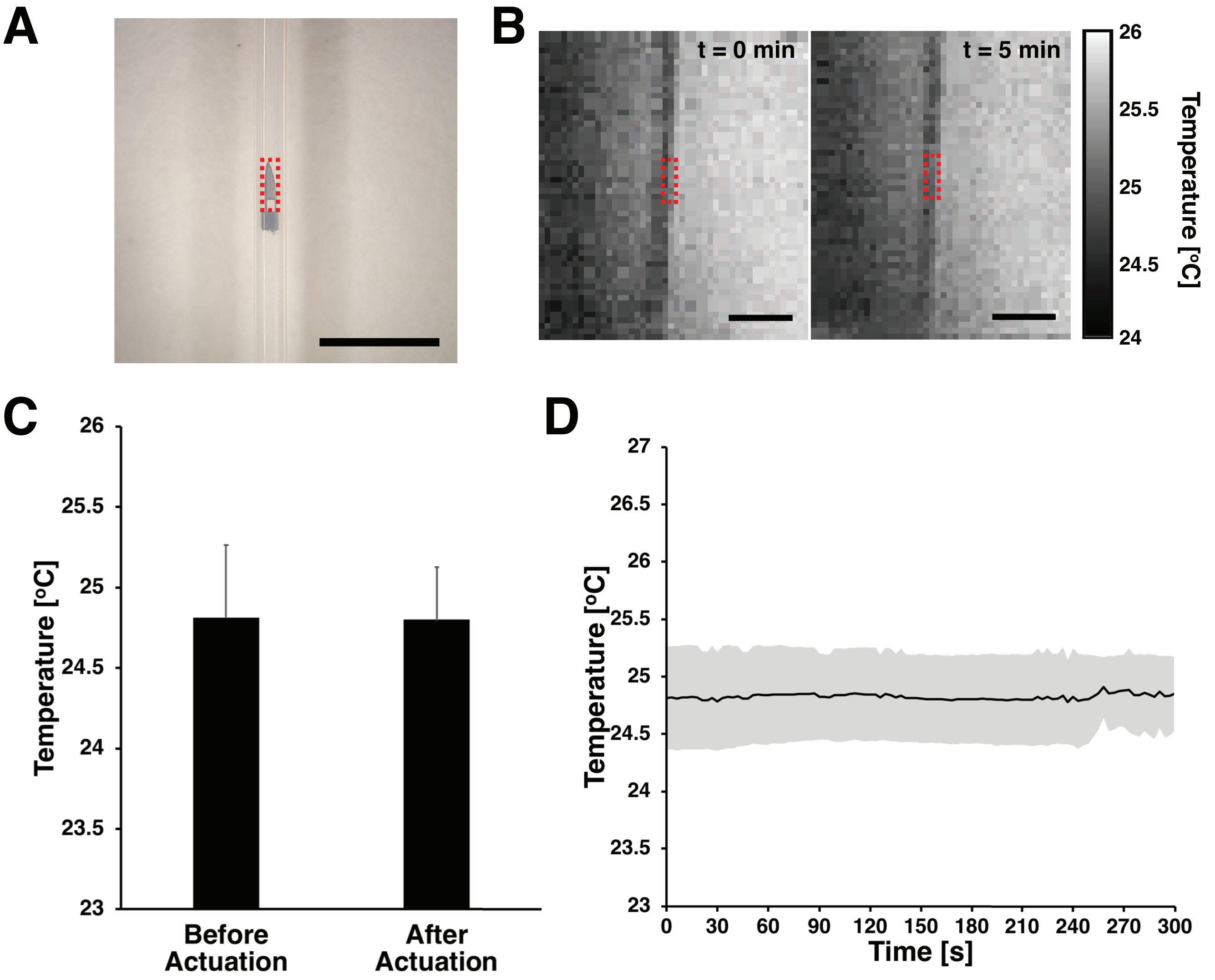

Figure 2. Temperature evaluations. (A) Photographs of a microactuator for temperature measurement. Red dotted box indicates the selected region of interest (ROI) for temperature analysis. Scale bar = 2 mm. (B) The infrared images of the ROI (the dotted red boxes) before actuation and after 5 min actuation. Scale bar = 2 mm. (C) Averaged temperature from the ROI before and after actuation (n = 4). (D) Averaged temperature from the ROI as a function of time with grey shaded error range.

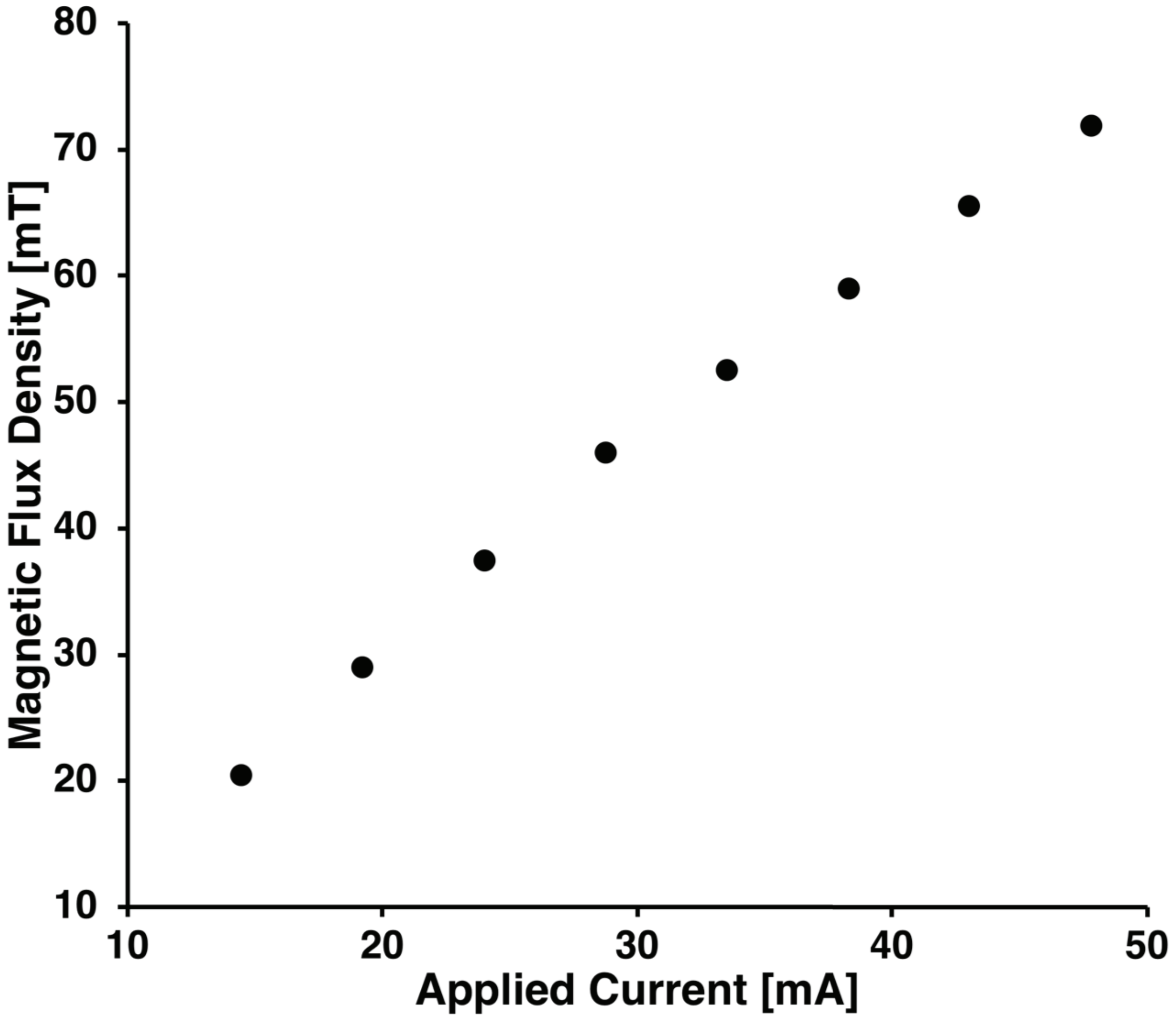

Figure 3. Magnetic field versus current. The amount of magnetic flux density required to deflect the microactuators from 8° to 64° ranged 10 to 40 mT.

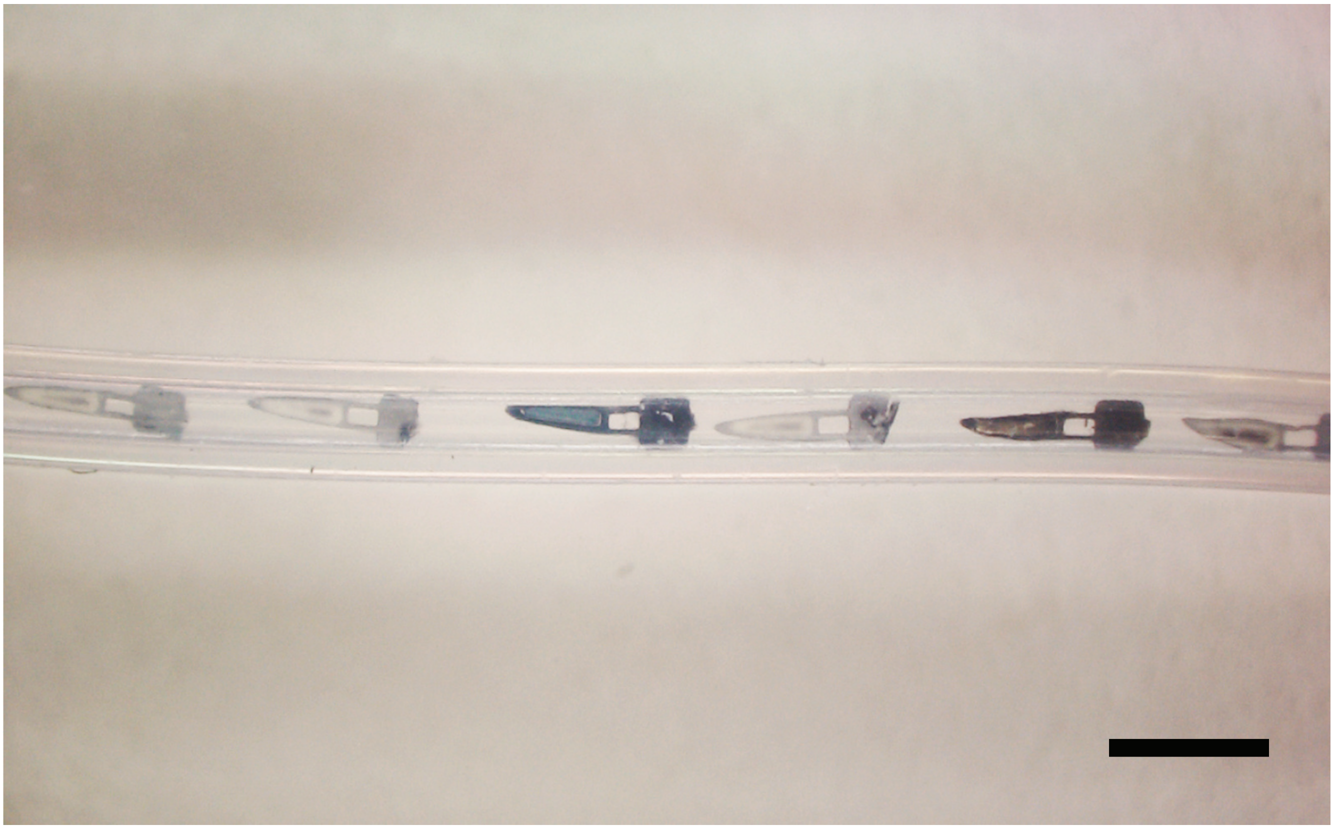

Figure 4. Photographs of multiple microactuators assembled in a microtube. Scale bar = 1 cm.
